# MALDI-TOF mass spectrometry of saliva samples as a prognostic tool for COVID-19

**DOI:** 10.1080/20002297.2022.2043651

**Published:** 2022-02-27

**Authors:** Lucas C. Lazari, Rodrigo M. Zerbinati, Livia Rosa-Fernandes, Veronica Feijoli Santiago, Klaise F. Rosa, Claudia B. Angeli, Gabriela Schwab, Michelle Palmieri, Dmitry J. S. Sarmento, Claudio R. F. Marinho, Janete Dias Almeida, Kelvin To, Simone Giannecchini, Carsten Wrenger, Ester C. Sabino, Herculano Martinho, José A. L. Lindoso, Edison L. Durigon, Paulo H. Braz-Silva, Giuseppe Palmisano

**Affiliations:** a GlycoProteomics Laboratory, Department of Parasitology, ICB, University of São Paulo, São Paulo, Brazil; b Laboratory of Virology (LIM-52-HC-FMUSP), Institute of Tropical Medicine of São Paulo, School of Medicine, University of São Paulo, São Paulo, Brazil; c Laboratory of Experimental Immunoparasitology, Department of Parasitology, ICB, University of São Paulo, São Paulo, Brazil; d Department of Stomatology, School of Dentistry, University of São Paulo, São Paulo, Brazil; e Department of Biosciences and Oral Diagnosis, Institute of Science and Technology, São Paulo State University, São José dos Campos, Brazil; f State Key Laboratory for Emerging Infectious Diseases, Department of Microbiology, Carol Yu Centre for Infection, Li KaShing Faculty of Medicine of the University of Hong Kong, Hong Kong, Special Administrative Region, China; g Department of Experimental and Clinical Medicine, University of Florence, Florence, Italy; h Unit for Drug Discovery, Department of Parasitology, ICB, University of São Paulo, São Paulo, Brazil; i Institute of Tropical Medicine of São Paulo, School of Medicine, University of São Paulo, São Paulo, Brazil; j Centro de Ciencias Naturais e Humanas, Universidade Federal do ABC, Santo André, Brazil; k Institute of Infectious Diseases Emílio Ribas, São Paulo, Brazil; l Laboratory of Protozoology (LIM-49-HC-FMUSP), Institute of Tropical Medicine of São Paulo, School of Medicine, University of São Paulo, São Paulo, Brazil; m Department of Infectious Diseases, School of Medicine, University of São Paulo, São Paulo, Brazil; n Laboratory of Clinical and Molecular Virology, Department of Microbiology, ICB, University of São Paulo, São Paulo, Brazil

**Keywords:** Saliva, SARS-CoV-2, biomarkers, proteomics, prognosis

## Abstract

**Background:**

The SARS-CoV-2 infections are still imposing a great public health challenge despite the recent developments in vaccines and therapy. Searching for diagnostic and prognostic methods that are fast, low-cost and accurate are essential for disease control and patient recovery. The MALDI-TOF mass spectrometry technique is rapid, low cost and accurate when compared to other MS methods, thus its use is already reported in the literature for various applications, including microorganism identification, diagnosis and prognosis of diseases.

**Methods:**

Here we developed a prognostic method for COVID-19 using the proteomic profile of saliva samples submitted to MALDI-TOF and machine learning algorithms to train models for COVID-19 severity assessment.

**Results:**

We achieved an accuracy of 88.5%, specificity of 85% and sensitivity of 91.5% for classification between mild/moderate and severe conditions. When we tested the model performance in an independent dataset, we achieved an accuracy, sensitivity and specificity of 67.18, 52.17 and 75.60% respectively.

**Conclusion:**

Saliva is already reported to have high inter-sample variation; however, our results demonstrates that this approach has the potential to be a prognostic method for COVID-19. Additionally, the technology used is already available in several clinics, facilitating the implementation of the method. Further investigation using a larger dataset is necessary to consolidate the technique.

## Introduction

Since its emergence in Wuhan in December 2019, the virus responsible for the Coronavirus Disease-2019 (COVID-19), SARS-CoV-2, has spread worldwide and become a world-threatening disease [1]. According to the World Health Organization (WHO), by the 17th of August of 2021 the COVID-19 pandemic reached a total of cumulative cases of 206,714,291 and cumulative deaths of 4,353,434 people worldwide [2]. Although many advances have been made in COVID-19 research, especially with the vaccines which initial data indicate their use was efficient and safe [^3–5^], the emergence of SARS-CoV-2 variants with the increased transmission is concerning [6]. Thus, searching for new diagnostic and prognosis methods is still needed for increasing patient survival.

Regarding prognosis and diagnostic methods, simplicity, speed, low cost and accuracy are desirable qualities. Diagnostic methods for COVID-19 are present commonly in two forms, immunological assays or RT-qPCR; the former is a fast and cheap method, but can be very limited for early diagnosis since the immune response is still forming; the latter is considered the ‘gold standard’ method and is the most used technique for COVID-19 diagnosis and disease tracking. However, the results are dependent of many factors, including proper sampling procedures and high-quality extraction kits [7,8]. Variations in these methods have been implemented to achieve more accurate, affordable, easy to use and scalable diagnostic platforms [9,10]. For being a relatively cheap and fast technique, MALDI-MS and machine learning algorithms have been implemented in many protocols, such as diagnosis and prognosis of several types of cancer [^11–13^], fungal and bacterial identification [14,15], detection of resistant fungi and bacteria [^16–18^], and COVID-19 diagnosis and prognosis [^19–23^]. However, these works used mainly plasma, serum or nasal swab samples, while saliva samples are still poorly explored. The process for collecting saliva is simple, fast, and painless and requires minimum supervision [24]. Saliva also contains high amounts of SARS-CoV-2 during the early stages of viral infection [1] and a recent study demonstrated that the salivary glands serve as a viral reservoir [25]; thus, this biofluid can be useful as source material for diagnosis and prognosis of COVID-19. Taken together, the rapid turnaround time (typically 10 min), high accuracy (> 95%) for detection of extremely low concentrations of biomolecules, low cost in supplies and technical processing indicates MALDI-MS as an attractive option of choice in COVID-19 diagnosis and prognosis.

In this work, we propose a new method for COVID-19 diagnosis and prognosis using the MALDI-TOF proteomic profile of salivary samples and machine learning. Samples from patients in a mild/moderate or severe conditions were used to train several machine learning algorithms and build a model to classify the samples into mild/moderate or severe COVID-19 cases. Moreover, another model was built to classify between infected and control samples. Thus, two models were studied one for diagnosis and one for prognosis, using a rapid and widespread technique, MALDI-TOF, which is common in clinical laboratories. Further investigations and a larger cohort are necessary to consolidate this technique as an alternative test for COVID-19 diagnosis or prognosis.

## Materials and methods

### Materials

All reagents used were from Sigma Aldrich unless otherwise stated.

### Ethical statement

This study was conducted in accordance with the Declaration of Helsinki, and the protocol was approved by the Research Ethics Committee of the ‘Instituto de Infectologia Emílio Ribas’, São Paulo, Brazil, protocol number CAAE 35589320.6.0000.0061. The invited volunteers were informed about the objectives, propositions and conditions of this project, in which those who agreed to participate in the research signed a free and informed consent term. Demographics, clinical data and samples were collected uniquely after the understanding of the study protocol and consent acknowledgement by the participants. A questionnaire on the health status of each participant was carried out. All participant information and samples were anonymized before use. Sample handling was carried out in a BSL2 laboratory.

### Individuals’ recruitment

COVID-19 infected-patients were classified in three groups according to the severity of the disease: 1) Mild form, characterized by the presence of influenza-like symptoms, with the absence of dyspnoea and normal radiological examination. 2) Moderate, characterized by the presence of influenza-like symptoms associated with pulmonary impairment < 50%, measured by computed tomography and O_2_ saturation > 93% in room air. 3) Severe characterized by respiratory frequency greater than 30 breaths per minute, O_2_ saturation < 93% in room air and pulmonary impairment > 50% measured by computed tomography. During the period from January 13 to 28 May 2021patients attending at the Instituto de Infectologia Emílio Ribas, Sao Paulo, Brazil, that tested molecularly positive for SARS-CoV-2 by a nasopharyngeal swab were invited to enroll in the research study and provide saliva samples on the day of inclusion. Individuals under 18 years old and pregnant women were excluded.

Healthcare workers group (controls). Asymptomatic healthcare workers potentially exposed to patients or SARS-CoV-2 positive samples were invited to enrol into the study. Saliva collection was performed on the day of individual inclusion and tested negative for SARS-CoV-2 by RT-PCR.

### COVID-19 routine diagnostic method (RT-PCR)

A standard protocol was used for nasopharyngeal swabs. A routine diagnosis protocol was applied for SARS-CoV-2 detection by RT-PCR assay, based on two distinct N gene targets, according to the protocol described by CDC (Center for Disease Control and Prevention, USA) (http//www.cdc.gov/coronavirus/2019-ncov/lab/rt-pcrp-panel-primer-probes.html). The limit of detection was 250 copies/mL.

### Saliva sample preparation

Saliva samples were obtained from SARS-CoV-2 infected and healthy individuals by using a cotton pad device – Salivette™ (Sarstedt AG & CO. KG, Nümbrecht, Germany). The patients were instructed not to drink, eat or tooth brush 2 hbefore sample collection. All the saliva samples were collected during the morning (from 7 AM to 11 AM). The patients were asked to maintain the cotton in the mouth for 90s; then, it was centrifuged at 1,000 g for 5 min. For patients under mechanical ventilation, a dentist, by using a tweezer, maintained the cotton pad in the oral cavity of the patient for 90s. After centrifugation, saliva samples were aliquoted and stored at −80°C. For viral inactivation, a solution of trifluoroacetic acid (TFA) 10% was added to the samples to obtain a final concentration of 1% of TFA in a BSL3 laboratory under all the safety measures needed. Then, protein quantification was performed using a Qubit assay (Thermo Fischer).

Different protein concentrations were evaluated before submitting the samples to MALDI-TOF analysis. Saliva samples were diluted with TFA 0.1% in the proportions of 1:1, 1:2, 1:5, 1:10 and 1:100; then, the samples were spotted directly in the MALDI target plate (Bruker Daltonics) using 1 μL of sample followed by 1 μL of matrix solution. Three different matrixes were also tested (sinapinic acid [SA], dihydroxybenzoic acid [DHB], and α-cyano-hydroxycinnamic acid [HCCA]) and the matrix solution was prepared by dissolving in acetonitrile/water 50:50 vol/vol containing 2.5% TFA to obtain a concentration of 10 mg/mL.

Additionally, we tested if adding the same concentration of proteins for each sample in the MALDI plate would be better for group separation. For that, the protein quantification of all saliva samples was performed and a total of 0.2 μg of proteins (protein normalization) were added to the MALDI plate, this was compared with the direct spotting of the samples without taking into consideration the protein quantification.

### MALDI acquisition

Samples were analyzed in a MALDI-TOF Autoflex speed smartbeam mass spectrometer (Bruker Daltonics, Bremen, Germany) using the FlexControl software (version 3.3, Bruker Daltonics). Spectra were recorded in the positive linear mode (laser frequency, 500 Hz; extraction delay time, 390 ns; ion source 1 voltage, 19.5 kV; ion source 2 voltage, 18.4 kV; lens voltage, 8.5 kV; mass range, 2,400 to 20,000 Da). Spectra were acquired using the automatic run mode to avoid subjective interference with the data acquisition. For each sample, 2,500 shots, in 500-shot steps, were summed. All spectra were calibrated by using the Protein Calibration Standard I (Insulin [M + H]+ = 5734.52, Cytochrome C [M + 2 H]2+ = 6181.05, Myoglobin [M + 2 H]2+ = 8476.66, Ubiquitin I [M + H]+ = 8565.76, Cytochrome C [M + H]+ = 12 360.97, Myoglobin [M + H]+ = 16 952.31) (Bruker Daltonics).

### Spectra processing

Data preprocessing and spectra evaluation were conducted using R packages. First, fid files were converted to mzML using MSconvert available in ProteoWizard (version: 3.0.20220) [26]. The mzML files were loaded to R using MALDiquantForeign and processed using MALDIquant [27]. The spectra for each sample were transformed (square root), smoothed (Savitzky-Golay and halfWindowSize of 10) [28] and the base line corrected using the TopHat algorithm [29]. Then, the intensities were normalized using the total ion current and the peaks were selected with a signal-to-noise ratio of 2 and halfWindowSize of 10 [22]. Peaks binning (tolerance of 0.003) and peak filtering (minimum frequency of 0.6) were performed for each group separately; then, a final binning (tolerance of 0.003) was performed with the groups merged. The resultant peaks were used to build an intensity matrix, which was further used for normality assessment (Shapiro-Wilk) and for a two-tailed Wilcoxon rank sum test corrected for multiple hypothesis testing using Benjamini-Hochberg. The peaks with a p-value < 0.05 were selected. The dataset was permuted 100 times and the global false discovery rate was calculated. For feature selection, the information gain method was used (FSelector package), since it is faster than wrapper methods and is classifier independent [30]. Features with weight higher than 0 were selected.

### Machine learning

Six different algorithms were tested (Support Vector Machine Polynomial – SVM-P, Gradient Boost Machine – GBM, Support Vector Machine Radial – SVM-R, Neural Networks – NNET, Naïve Bayes – NB, and Random Forest – RF) to classify samples from patients in severe or mild/moderate and infected or control conditions. The training was carried through fourfold nested repeated ten times four-fold cross-validation using the Caret package. The data were split randomly. A random search was performed for hyperparameter tunning in the inner loop. The MLeval package was used to plot the Receiver Operating Characteristic (ROC) and Precision Recall (PR) curves. The area under the curve (AUC) of the ROC curves, accuracy, sensitivity and specificity were reported. Since the infected and control groups were not balanced, the balanced accuracy was used. For the prognosis machine learning (severe versus mild/moderate) we calculated the brier score for each fold.

**Figure 1.**
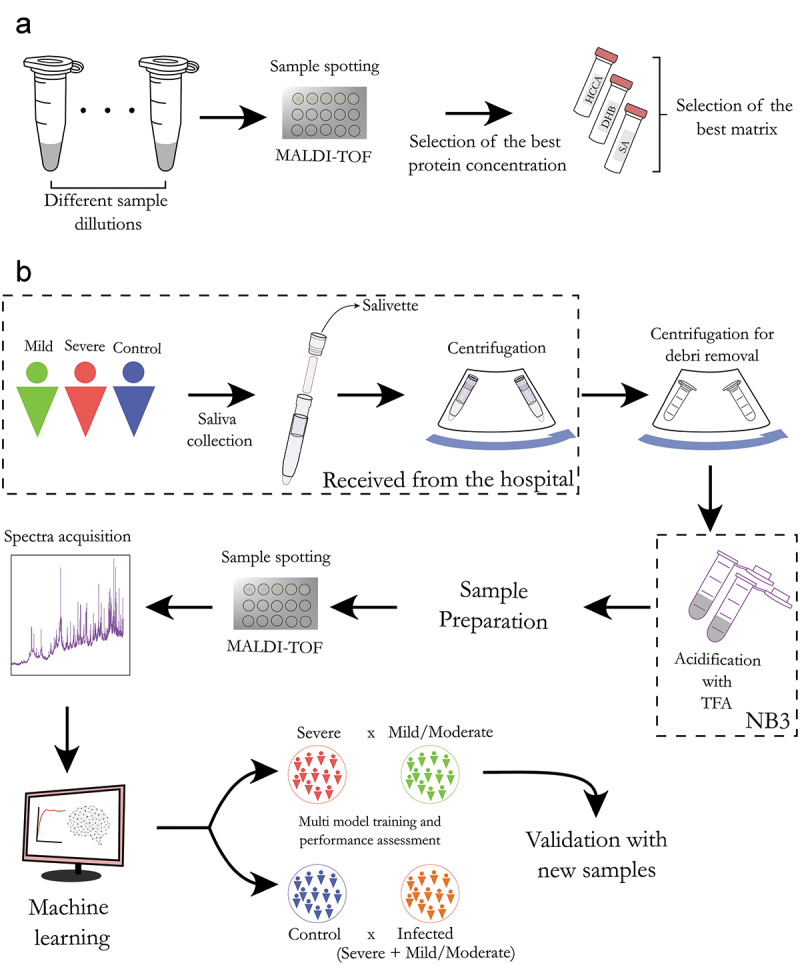
General workflow of the saliva sample preparation and MALDI-TOF analysis. (a) Method optimization. Initially, a range of different protein concentrations was evaluated to determine the optimal protein concentration. Subsequently, three MALDI matrices with better performances were evaluated and α-cyano-hydroxycinnamic acid (HCCA) was selected as the optimal one. (b) The sample cohort consisted of four groups: severe (ICU), mild (emergency room), moderate (department) and control. The COVID-19 diagnosis was confirmed by RT-qPCR of nasopharyngeal swabs. Saliva samples were obtained using a salivette and virus inactivation was performed in an BSL-3 laboratory. Direct spotting of saliva on the MALDI plate was performed and the proteomic profile was acquired using a MALDI-TOF MS instrument. The MALDI-TOF spectra were pre-processed and analyzed in R environment, where statistically significant protein peaks were used to train multiple machine learning algorithms for group classification (training phase). The model with the best performances was used to analyze independent samples and calculate the accuracy, sensitivity and specificity of the model (validation phase).

For the classification between severe and mild/moderate, the model with the best performance was selected for a validation step using another set of samples (test set) prepared and acquired separately from the training samples. The same preprocessing was carried out for the test set samples separately from the training samples. However, train and test sets were binned together to generate comparable features. SVM-R demonstrated the best performance in the algorithm comparison step; thus, it was selected for model training in the validation step. The parameters were fixed at sigma = 0.003329177 and C = 0.8915975, since they were the parameters that achieved the best accuracy for SVM-R. The model training was conducted in two ways; (1) The training set was used directly for model training, then, the test set was applied to the model and the metrics were calculated; (2) an extra pre-processing step was performed in the training set prior to model training. We conducted a feature selection using the information gain algorithm (FSelector) to select the features with higher importance (weights higher than zero). After feature selection, the model was trained using only the selected features, then the test set was applied to evaluate the model performance. A schematic workflow is presented in Figure 1.

## Results

In this study, we evaluated the potential of MALDI-TOF to identify prognostic biomarkers using saliva of COVID-19 patients. Initially, we tested a combination of analytical parameters to find the optimal conditions to obtain reliable and reproducible MS profiles (Figure 1). These conditions were optimized using a subset of samples belonging to the COVID-19 cohort. The entire cohort contained a total of 196 patients diagnosed with COVID-19, being 42 (21.4%) evaluated in emergency rooms, 81 (41.3%) hospitalized and 73 (37.3%) in the intensive care unit (ICU), and 36 controls. Within the COVID-19 patients, we classified the emergency room (outpatient and inpatient) patients as in a MILD/MODERATE condition and ICU patients as in a SEVERE condition. Most patients were male with an average age of 52.4 years old, being 153 (78%) patients more than 40 years old. Most of them were not vaccinated (172, 87.7%) and used non-invasive oxygen support (106, 54%), 23 patients (11.7%) deceased due to COVID-19 complications. Most ICU patients were male and more than 40 years old, all patients with invasive oxygen support and 20 of the 23 death outcome patients were admitted to the ICU. Sex (p = 0.003), oxygen support type (p < 0.001) and outcome (p < 0.001) showed statistical significance between emergency room/department and ICU. The most prevalent clinical symptoms were cough (67.9%), fever (61.7%), breathing difficulty (58.2%), fatigue (40.8%) and body or muscular aches (37.8%). We observed that sex (p = 0.003), mechanical ventilation (p < 0.001), body or muscular aches (p = 0.046) and breathing difficulty (p = 0.004) were statistically significant different between the emergency room/department and ICU, being the latter being the most prevalent in the patients treated in emergency rooms or hospitalized, and breathing difficulty most prevalent in ICU patients (Table 1). No statistical difference in sex and lower age for the controls was detected between COVID-19 patients and control subjects (Supplementary Table S1).

**Table 1.** Clinical characteristics of the 192 COVID-19 patients investigated

	MILD/MODERATE		SEVERE		Total		p^(a)^
Patients characteristics	N	%	N	%	N	%	
Sex							
Male	59	53.6%	51	46.5%	110	100%	0.003*
Female	64	74.4%	22	25.6%	86	100%	
Age							
<40	30	69.8%	13	30.2%	43	100%	0.282
≥40	93	60.8%	60	39.2%	153	100%	
Elderly							
<60	88	65.7%	46	34.3%	134	100%	0.214
≥60	35	56.5%	27	43.5%	62	100%	
Clinical features							
Mechanical ventilation							
None	59	96,7%	2	3,3%	61	100%	<0.001*
Non-invasive^a^	64	60,4%	42	39,6%	106	100%	
Invasive^b^	0	0,0%	29	100,0%	29	100%	
Vaccination status							
Unvaccinated	106	61.6%	66	38.4%	172	100%	0.588
First dose only	10	66.7%	5	33.3%	15	100%	
Fully vaccinated	7	77.8%	2	22.2%	9	100%	
Outcome							
Discharge	120	69.4%	53	30.6%	173	100%	<0.001*
Death	3	13.0%	20	87.0%	23	100%	
Prevalence of symptoms according to disease severity							
Fever	80/123	65.0%	41/73	56.2%	121/196	61.7%	0.216
Cough	80/123	65.0%	53/73	72.6%	133/196	67.9%	0.273
Headache	51/123	41.5%	21/73	28.8%	72/196	36.7%	0.075
Sore throat	28/123	22.8%	11/73	15.1%	39/196	19.9%	0.192
Muscle or body aches	53/123	43.1%	21/73	28.8%	74/196	37.8%	0.046*
Fatigue	52/123	42.3%	28/73	38.4%	80/196	40.8%	0.589
Congestion or runny nose	23/123	18.7%	9/73	12.3%	32/196	16.3%	0.243
Difficulty breathing	62/123	50.4%	52/73	71.2%	114/196	58.2%	0.004*
Loss of smell	19/123	15.4%	9/73	12.3%	28/196	14.3%	0.546
Loss of taste	21/123	17.1%	17/73	23.3%	38/196	19.4%	0.287
Nausea or vomiting	22/123	17.9%	13/73	17.8%	35/196	17.9%	0.989
Diarrhea	21/123	17.1%	8/73	11.0%	29/196	14.8%	0.244

ICU – Intensive Care Unit; *Statistical significance; (1) Pearson Chi-Square.

^a^Nasal cannula, facial mask ventilation or high flow nasal cannula.

^b^Invasive mechanical ventilation.

**Figure 2.**
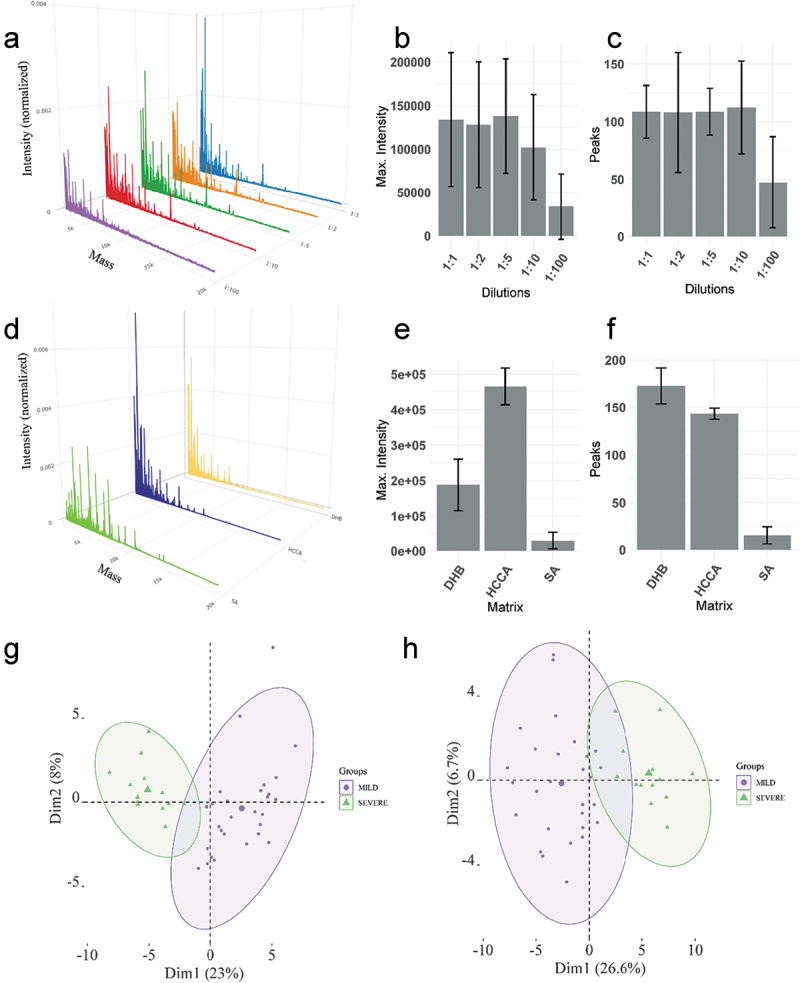
(a) Mean spectra profile obtained from three different samples in triplicate at different concentrations (1:1, 1:2, 1:5, 1:10 and 1:100 sample to TFA 0.1% ratio). (b) Maximum intensity of the spectra for each concentration. (c) The number of peaks identified for each concentration. (d) Mean spectra profile of one sample in triplicate for each matrix tested. (e) Maximum intensity of the spectra for each matrix. (f) Number of peaks detected for each matrix. (g) PCA of the samples that were analyzed with 0.2 μg of proteins. (h) PCA of the samples using 1ul of saliva irrespective of the protein concentration. In Figure 2(g,h), MILD refers to MILD/MODERATE condition.

The optimization step consisted in testing several approaches for spectra acquisition. Initially, we tested the direct spotting method using several dilutions of the saliva. Three saliva samples were randomly selected from the cohort and analyzed in technical triplicates. We observed that direct spotting of samples diluted in a proportion of 1:1, 1:2, 1:5 and 1:10 presented similar number of peaks and maximum intensity, as well as a similar spectra profile (Figure 2). Thus, we selected the dilution of 1:10 that corresponded to a protein concentration of approximately 0.2 μg/μL to conduct the following optimization step. The selection of the optimal MALDI matrix was performed using the same saliva sample in technical triplicate. Three MALDI matrices, sinapinic acid [SA], dihydroxybenzoic acid [DHB], and α-cyano-hydroxycinnamic acid [HCCA] were tested and their MS profile compared. Different spectra profiles were obtained, being HCCA with higher maximum intensity, and DHB with more peaks identified (Figure 2). Although DHB had more peaks, we selected HCCA for the next analyses since it had lower variation in terms of number of peaks and maximum intensity. From these data it was possible to notice that analyses of technical replicates of the same saliva sample lead to less variation compared to different saliva samples (Figure 2(b–c vs 2(e–f)).

A small portion of the dataset (87 samples) was used to test whether loading the same amount of proteins in the MALDI plate would be better for group separation. We observed that loading 0.2 μg of proteins for each sample reduced the variation in the PCAs (Figure 2g,h). Thus, for the machine learning analysis we decided to conduct sample spotting of the entire dataset using 0.2 μg of proteins. The variation obtained between MILD/MODERATE and SEVERE was higher compared to the variation obtained within the same condition.

**Figure 3.**
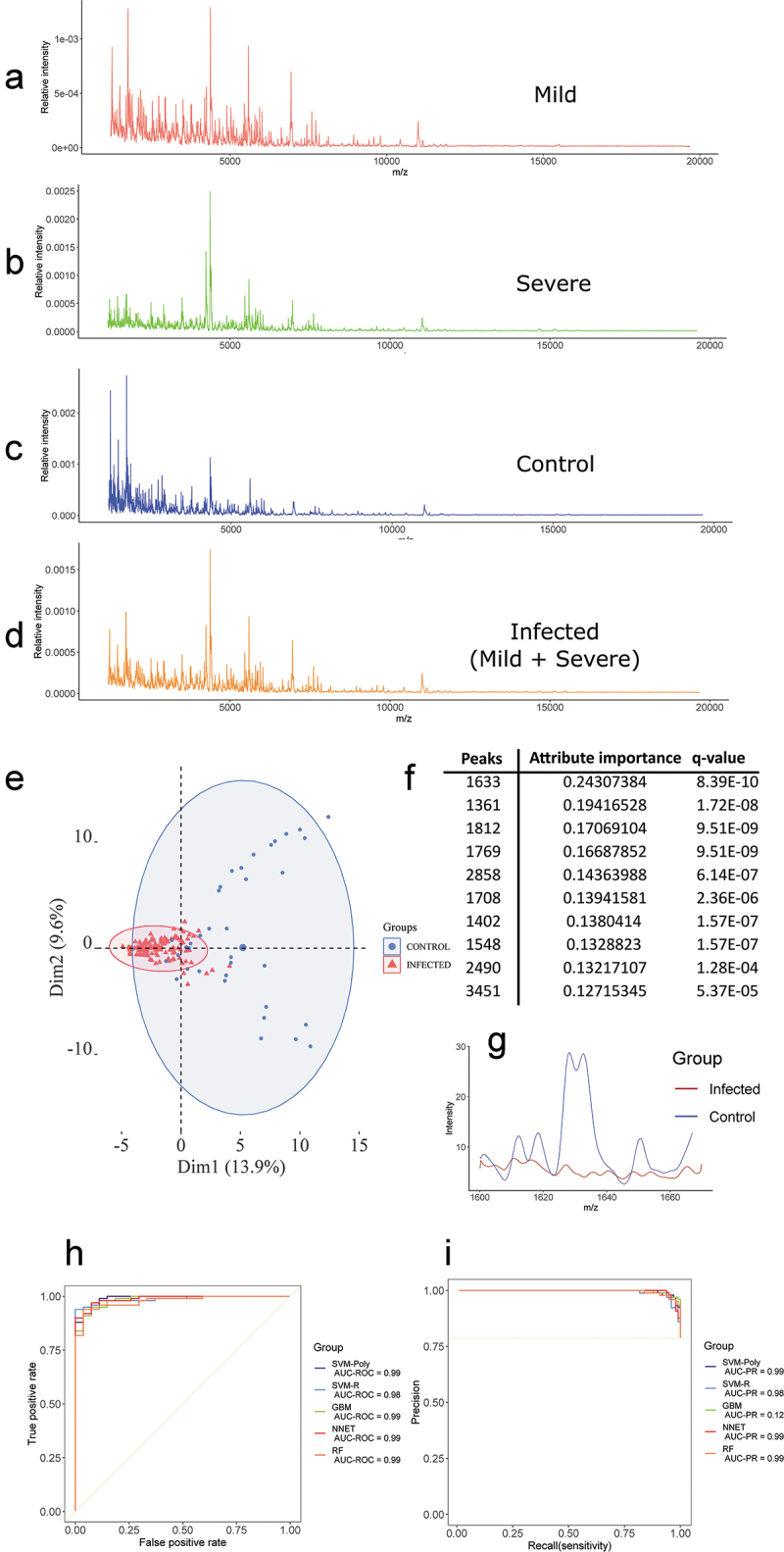
(a) MALDI-TOF MS mean spectrum of the saliva of the MILD/MODERATE group. (b) MALDI-TOF MS mean spectrum of the saliva of the SEVERE group. (c) MALDI-TOF MS mean spectrum of the saliva of the control group. (d) MALDI-TOF MS mean spectrum of the saliva of the infected group (MILD/MODERATE+SEVERE). (e) PCA for the control and infected groups. (f) The most ranked peaks after information gain filtering. (g) Spectra from the region of the highest ranked peak (1633). (h) Best ROC curves obtained from model training with control and infected samples. (i) Best PR curves obtained from model training with control and infected samples.

This study was divided into two parts comparing the saliva samples of: 1) control versus COVID-19 infected patients and 2) MILD/MODERATE versus SEVERE cases of COVID-19 patients. Average spectra for the control (36 samples), MILD/MODERATE (51 samples), SEVERE (81 samples) and MILD/MODERATE+SEVERE (132 samples) are reported in Figure 3(a–d).

Initially, we aimed to compare the MALDI-TOF MS profiles of control versus infected patients. A total of 132 samples of COVID-19 patients combining MILD, MODERATE and SEVERE were used as the infected group and 36 control samples were used as the control group. After peak filtration using the MALDIquant package, a total of 183 peaks were identified, which were reduced to 99 peaks after normality assessment and Wilcoxon rank sum test. The remaining peaks were used to plot the PCAs (Figure 3(e)) and train six machine learning models to classify between the conditions. The values for balanced accuracy and specificity were high for all models (Supplementary Table S2), with the NB model having lower values. The sensitivity was high for SVM-P, SVM-R and RF, and balanced accuracy measures were similar for SVM-P and SVM-R, both with the highest value amongst the models tested. The complete list of all metrics obtained and all optimized hyperparameters for each fold are available at Supplementary Tables S3 and S4. Overall, the best model was SVM-P, which scored a balanced accuracy, sensitivity and specificity of 92.9%, 92.2% and 93.6%. The 99 statistically significant features (q value less than 0.05) were used to train the model (Figure 3(h–i)) and Supplementary Table S5). Additionally, we performed an Information Gain filtering of the most relevant peaks and selected the 10 most ranked ones (Figure 3(f)) and plotted the region of the most ranked peak (Figure 3(g)).

These data showed a pronounced difference between the two conditions. This prompted us to investigate the MS profile of saliva samples from COVID-19 patients harboring different clinical characteristics.

**Figure 4.**
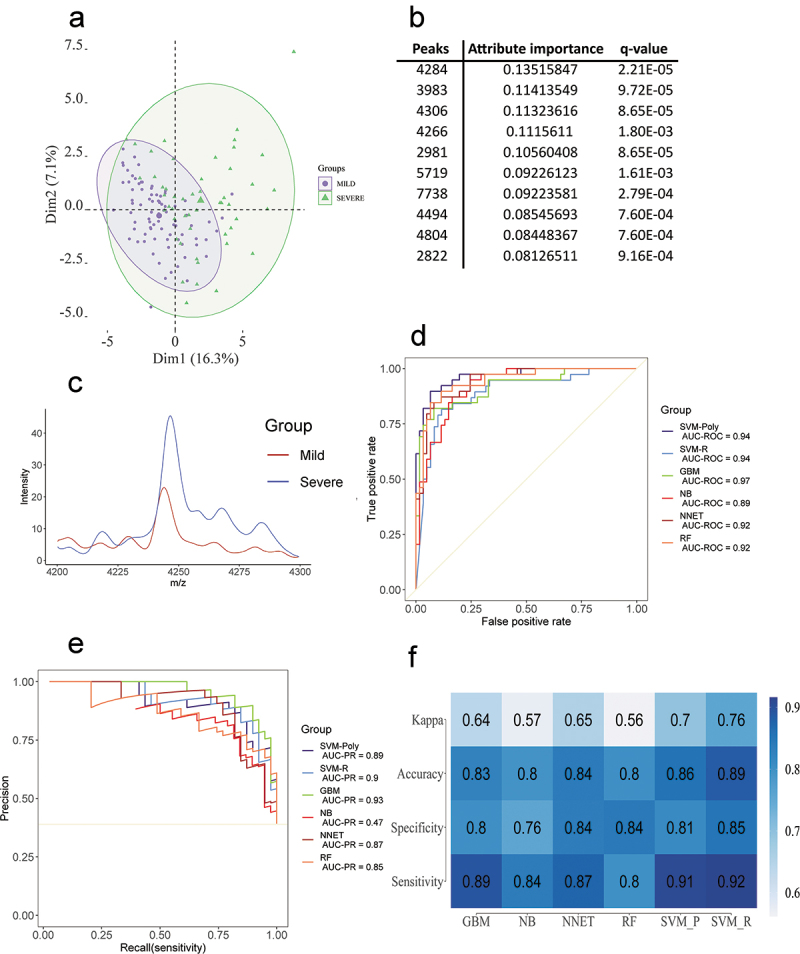
(a) PCA obtained through the analysis of the MILD/MODERATE and SEVERE conditions. (b) The 10 most relevant peaks were obtained through information gain filtering. (c) Comparison between MILD/MODERATE and SEVERE of the most discriminant peak. (d) Best ROC curves obtained from the model training. (e) Best PR curves obtained from the model training. (f) Mean accuracy, mean sensitivity, mean specificity and mean kappa were obtained in the model training step.

Due to that, we analyzed 132 samples of COVID-19 patients (67% of the total samples) divided in 81 MILD/MODERATE and 51 SEVERE to identify MS-based saliva features. After peak filtration using the MALDIquant package, a total of 139 peaks were identified, which were reduced to 44 peaks after normality assessment and Wilcoxon rank sum test. The remaining peaks were used to plot the PCA (Figure 4(a)) and train six machine learning models to classify between the conditions. The performance metrics for each model indicated that they differed, being the SVM-R as the model with best mean accuracy (88.5%), mean sensitivity (91.5%) and mean specificity (85%) (Figure 4(f) and Supplementary Table S6). Also, specificity presented a high variation in every model tested, which was expected, since the saliva samples already presented a high variation in the PCA (Figure 4(a)). This was not observed in our previous study using the same platform but plasma as biofluid [23]. The performance metrics in each fold for all six models, the optimized hyperparameters and brier scores are available in the Supplementary Tables S7–S9. The best ROC and PR curves for each model are presented in the Figure 4(d–e). The 44 statistically significant features (q value less than 0.05) were used to train the model (Figure 4(d) and Supplementary Table S10). Additionally, we performed a feature selection by information gained to observe which were the most relevant peaks, the 10 most important peaks are reported in Figure 4(b) and the most rated peak is plotted in Figure 4(c).

For SEVERE and MILD/MODERATE classification model validation, we prepared extra 64 samples (33% of the total samples) to test if the model could classify them correctly. We trained the SVR-R algorithm using optimized parameters (sigma = 0.003329177 and C = 0.8915975) retrieved from the model selection step. First, we applied the test set to the trained model without feature selection, this approach yielded an accuracy of 67.18%, a sensitivity of 52.17% and a specificity of 75.60%. Then, we tested whether training the model using features that were selected by the information gain method could generate a better result; however, the metrics obtained were slightly lower, with an accuracy of 65.62%, sensitivity of 50% and specificity of 70.83%. The values obtained are expected since we used an independent dataset to validate the model generated during the training step. Since the MALDI-TOF MS features were extracted from the highly variable saliva samples, it was expected we would to retrieve lower accuracy, sensitivity and specificity values.

## Discussion

The use of machine learning to build models for COVID-19 diagnosis has been already reported in the literature. Several approaches have been tested, including the use of X-ray imaging data [31], emergency care admission examinations [32] and routine blood tests [33]. Also, mass spectrometry data have been used to train machine learning algorithms. Among the biofluids used and the mass spectrometry techniques performed are analysis of nasopharyngeal swabs in the MALDI-TOF spectrometer to build models for the COVID-19 diagnosis [22,34], the analysis of proteomic profile of plasma samples by LC-MS/MS to build models for severity assessment and drug repurposing analysis [35], the use of the metabolomic profile obtained from plasma samples submitted to direct injection in the high resolution MS system to build models for COVID-19 diagnosis and risk assessment [21], and the use of the proteomic profile of plasma samples submitted to MALDI-TOF to build models for COVID-19 risk assessment [23]. Many of the studies are performed using biofluids that are invasive, such as plasma and nasopharyngeal swabs, and uses data acquisition techniques that are relatively expensive and time demanding. In order to slow down the disease spread, rapid and accurate identification of SARS-CoV-2 is necessary [36]; thus, we describe here the use of a minimal invasive biofluid associated with MALDI-TOF, which is a relatively cheap and fast technique, for the diagnosis and prognosis of COVID-19 using machine learning algorithms. Our models attained a high performance, with the best model achieving an accuracy, sensitivity and specificity of 88.5%, 91.5% and 85% respectively for classifying between mild/moderate and severe cases of COVID-19; also, we achieved a balanced accuracy, sensitivity and specificity of 92.9%, 92.2% and 93.6% respectively for classifying between infected and control samples.

Although the models demonstrated a good performance in the training steps, the validation with independent data presented a lower performance than expected. It is known that the salivary proteome is a complex biofluid, with reports indicating an identification of 5,500 proteins [37]. Also, there are several factors that contribute to inter-individual variability of the protein composition, such as taste stimulation, collection at different times of the day, age of infants, genetic polymorphism, and systemic pathologies [38]. Although the patients were instructed to not drink, eat or tooth brush 2 hbefore sample collection, it is important to note that these activities can alter the saliva composition, and thus must be controlled or documented during sample collection (doi: 10.7150/ijms.25146). Saliva collection and storage must also be taken into consideration since proteolytic activity occurs within 30 min after collection (PMID: 19578491), short-term storage in ice can reduce protein degradation and sample centrifugation is recommended to separate cells from proteins in the supernatant, since it is known that protein degradation in the supernatant is significantly slower (PMID: 21917601). Saliva collection was conducted in a standardized manner; however, it is extremely difficult to control the behavior of each individual prior to sample collection. The direct spotting of the saliva samples might be another factor that contributed to the variability observed. Generally, direct spotting is more common in the literature, but other sample preparation methods might be applied prior to spotting, such as protein digestion, glycoproteins enrichment, protein separation by electrophoretic gels, and peptide purification by C18 resins [^38–41^]. Optimization and standardization of the saliva collection and preparation are still being studied in the literature, but the use of Salivette tubes and direct spotting on the same day of collection were demonstrated to maintain intra-individual stability of the saliva MS profile (PMID: 34441239). Since our proposal was to develop the simplest yet accurate method for COVID-19 diagnosis, we did not evaluate the effect of more elaborate sample preparation methods; thus, further investigation is required in order to evaluate if the variability issue could be solved in the machine learning analysis.

We achieved a good accuracy for classifying between control and infected samples; however, it is important to note that our controls do not comprise samples obtained from patients that presented influenza symptoms, meaning that it is still necessary to evaluate if the models trained are specific for COVID-19. Comparing infected patients with controls that presented similar symptoms is commonly done in the literature [19,20,22]; however, since our study was aimed at prognosis, there were few control samples available in our cohort and all of them were from healthy patients. A study by Costa et al. (2021) demonstrated the potential of MALDI-TOF analysis of saliva samples and machine learning for COVID-19 diagnosis, they also reported high inter-individual variability and their models did not achieve sensitivity, specificity and accuracy values higher than 85% [42]; our approach demonstrated a potential application for prognosis, and our models achieved similar performances, meaning that the results corroborates with what is expected for saliva samples.

This study demonstrates that MALDI-TOF MS and machine learning algorithms can be used to analyze saliva samples for prognosis purposes. This strategy is reproducible, easy to perform, fast, and low-cost. Since MALDI-TOF MS is already present in several clinical laboratories, this approach can be easily established in hospitals. However, it is important to recognize the limitations of this technique. We observed high inter-sample variation, which can reduce the performance of the models trained and reduce even more the performance during model generalization (validation with unseen data). This could be minimized by adding more samples to the cohort or by using more elaborate sample preparation methods. Therefore, a larger cohort should be analyzed to develop more robust models and inter-laboratory samples should be used to validate the findings. Moreover, improvements in the analytical part should be able to discriminate better between MILD and MODERATE groups.

## Supplementary Material

Supplemental Material

## References

[1] Aydin S, Benk IG, Geckil AA. May viral load detected in saliva in the early stages of infection be a prognostic indicator in COVID-19 patients? J Virol Methods. 2021;294:114198. 34044003 10.1016/j.jviromet.2021.114198 PMC8142821

[2] World Health Organization. COVID-19 weekly epidemiological update. World Heal Organ. 2021;1–12.

[3] Daniel W, Nivet M, Warner J, et al. Early evidence of the effect of SARS-CoV-2 vaccine at one medical center. N Engl J Med. 2021;384:1962–1963. 33755374 10.1056/NEJMc2102153 PMC8008752

[4] Haas EJ, Angulo FJ, McLaughlin JM, et al. Impact and effectiveness of mRNA BNT162b2 vaccine against SARS-CoV-2 infections and COVID-19 cases, hospitalisations, and deaths following a nationwide vaccination campaign in Israel: an observational study using national surveillance data. Lancet. 2021;397:1819–1829. 33964222 10.1016/S0140-6736(21)00947-8 PMC8099315

[5] Shrotri M, Krutikov M, Palmer T, et al. Vaccine effectiveness of the first dose of ChAdOx1 nCoV-19 and BNT162b2 against SARS-CoV-2 infection in residents of long-term care facilities in England (VIVALDI): a prospective cohort study. Lancet Infect Dis. 2021;3099:1–10. 10.1016/S1473-3099(21)00289-9 PMC8221738 34174193

[6] Fontanet A, Autran B, Lina B, et al. SARS-CoV-2 variants and ending the COVID-19 pandemic. Lancet. 2021;397:952–954. 33581803 10.1016/S0140-6736(21)00370-6 PMC7906631

[7] Mathuria JP, Yadav R, Rajkumar. Laboratory diagnosis of SARS-CoV-2 - A review of current methods. J Infect Public Health. 2020;13:901–905. 32534946 10.1016/j.jiph.2020.06.005 PMC7275982

[8] Tahamtan A, Ardebili A. Real-time RT-PCR in COVID-19 detection: issues affecting the results. Expert Rev Mol Diagn. 2020;20:453–454. 32297805 10.1080/14737159.2020.1757437 PMC7189409

[9] Falzone L, Gattuso G, Tsatsakis A, et al. Current and innovative methods for the diagnosis of COVID-19 infection (Review). Int J Mol Med. 2021;47:1–23. 10.3892/ijmm.2021.4933 PMC8043662 33846767

[10] Oliveira BA, de Oliveira LC, Sabino EC, et al. SARS-CoV-2 and the COVID-19 disease: a mini review on diagnostic methods.Rev Inst Med Trop Sao Paulo. 2020;62:1–8. 10.1590/S1678-9946202062044 PMC7325591 32609256

[11] Pham P, Yu L, and Nguyen M. A novel algorithm for multi-class cancer diagnosis on MALDI-TOF mass spectra. In Proc. - 2011 IEEE Int. Conf. Bioinforma. Biomed. BIBM 2011. Atlanta, GA, USA; 2011. P. 398–401. doi:10.1109/BIBM.2011.50.

[12] Pan Y, Zhang L, Zhang R, et al. Screening and diagnosis of colorectal cancer and advanced adenoma by Bionic Glycome method and machine learning. American Journal of Cancer Research. 2021;11(6):3002–3020. 34249441 PMC8263652

[13] Buszewska-Forajta M, Pomastowski P, Monedeiro F, et al. Lipidomics as a diagnostic tool for prostate cancer. Cancers (Basel). 2021;13(9):1–20. 10.3390/cancers13092000 PMC8122642 33919225

[14] Cassagne C, Normand AC, L’Ollivier C, et al. Performance of MALDI-TOF MS platforms for fungal identification. Mycoses. 2016;59:678–690. 27061755 10.1111/myc.12506

[15] Desaire H, Hua D. Adaption of the aristotle classifier for accurately identifying highly similar bacteria analyzed by MALDI-TOF MS. Anal Chem. 2020;92:1050–1057. 31769656 10.1021/acs.analchem.9b04049 PMC7676635

[16] Tang K, Tang D, Wang Q, et al. MALDI-TOF MS platform combined with machine learning to establish a model for rapid identification of methicillin-resistant Staphylococcus aureus. J Microbiol Methods. 2021;180:106109. 33271209 10.1016/j.mimet.2020.106109

[17] Delavy M, Cerutti L, Croxatto A, et al. Machine learning approach for *Candida albicans* fluconazole resistance detection using matrix-assisted laser desorption/ionization time-of-flight mass spectrometry. Front Microbiol. 2020;10. DOI:10.3389/fmicb.2019.03000. PMC6971193 32010083

[18] Zvezdanova ME, Arroyo MJ, Méndez G, et al. Detection of azole resistance in Aspergillus fumigatus complex isolates using MALDI-TOF mass spectrometry. Clin Microbiol Infect. 2021. DOI:10.1016/j.cmi.2021.06.005. 34147673

[19] Yan L, Yi J, Huang C, et al. Rapid detection of COVID-19 using MALDI-TOF-based serum peptidome profiling. Anal Chem. 2021;93:4782–4787. 33656857 10.1021/acs.analchem.0c04590

[20] Deulofeu M, García-Cuesta E, Peña-Méndez EM, et al. Detection of SARS-CoV-2 infection in human nasopharyngeal samples by combining MALDI-TOF MS and artificial intelligence. Front Med. 2021;8:1–12. 10.3389/fmed.2021.661358 PMC8047105 33869258

[21] Delafiori J, Navarro LC, Siciliano RF, et al. Covid-19 automated diagnosis and risk assessment through metabolomics and machine learning. Anal Chem. 2021;93:2471–2479. 33471512 10.1021/acs.analchem.0c04497 PMC8023531

[22] Nachtigall FM, Pereira A, Trofymchuk OS, et al. Detection of SARS-CoV-2 in nasal swabs using MALDI-MS. Nat Biotechnol. 2020. DOI:10.1038/s41587-020-0644-7 32733106

[23] Lazari LC, Ghilardi FDR, Rosa-Fernandes L, et al. Prognostic accuracy of MALDI-TOF mass spectrometric analysis of plasma in COVID-19. Life Sci Alliance. 2021;4:1–12. 10.26508/lsa.202000946 PMC8321665 34168074

[24] Yee R, Truong TT, Pannaraj PS, et al. Saliva is a promising alternative specimen for the detection of SARS-CoV-2 in children and adults. J Clin Microbiol. 2021;59. DOI:10.1128/JCM.02686-20. PMC8111155 33239380

[25] Matuck BF, Dolhnikoff M, Duarte‐Neto AN, et al. Salivary glands are a target for SARS-CoV-2: a source for saliva contamination. J Pathol. 2021;254:239–243. 33834497 10.1002/path.5679 PMC8250228

[26] Matthew C, Maclean B, and Burke R, et al. A cross-platform toolkit for mass spectrometry and proteomics. Nat Biotechnol. 2012;30:1–8. 23051804 10.1038/nbt.2377 PMC3471674

[27] Gibb S, Strimmer K. Maldiquant: a versatile R package for the analysis of mass spectrometry data. Bioinformatics. 2012;28:2270–2271. 22796955 10.1093/bioinformatics/bts447

[28] Savitzky A, Golay MJE. Smoothing and differentiation of data by simplified least squares procedures. Anal Chem. 1964;36:1627–1639.

[29] Trapnell C, Pachter L, Salzberg SL. TopHat: discovering splice junctions with RNA-Seq. Bioinformatics. 2009;25:1105–1111. 19289445 10.1093/bioinformatics/btp120 PMC2672628

[30] Cheng T, Wang Y, Bryant SH. FSelector: a Ruby gem for feature selection. Bioinformatics. 2012;28:2851–2852. 22942017 10.1093/bioinformatics/bts528 PMC3476337

[31] Elaziz MA, Hosny KM, Salah A, et al. New machine learning method for imagebased diagnosis of COVID-19. PLoS One. 2020;15. DOI:10.1371/journal.pone.0235187. PMC7319603 32589673

[32] de Moraes Batista AF, Miraglia JL, Donato THR, et al. COVID-19 diagnosis prediction in emergency care patients: a machine learning approach. medRxiv. 2020. DOI:10.1101/2020.04.04.20052092

[33] Kukar M, Gunčar G, Vovko T, et al. COVID-19 diagnosis by routine blood tests using machine learning. Sci Rep. 2021;11:1–9. 34031483 10.1038/s41598-021-90265-9 PMC8144373

[34] Rocca MF, Zintgraff JC, Dattero ME, et al. A combined approach of MALDI-TOF mass spectrometry and multivariate analysis as a potential tool for the detection of SARS-CoV-2 virus in nasopharyngeal swabs. J Virol Methods. 2020;286. DOI:10.1016/j.jviromet.2020.113991. PMC7546642 33045283

[35] Suvarna K, Biswas D, Pai MGJ, et al. Proteomics and machine learning approaches reveal a set of prognostic markers for COVID-19 severity with drug repurposing potential. Front Physiol. 2021;12:1–18. 10.3389/fphys.2021.652799 PMC8120435 33995121

[36] Kumar R, Nagpal S, Kaushik S, et al. COVID-19 diagnostic approaches: different roads to the same destination. VirusDisease. 2020;31:97–105. 32656306 10.1007/s13337-020-00599-7 PMC7293170

[37] Grassl N, Kulak NA, Pichler G, et al. Ultra-deep and quantitative saliva proteome reveals dynamics of the oral microbiome. Genome Med. 2016;8:1–13. 27102203 10.1186/s13073-016-0293-0 PMC4841045

[38] Quintana M, Palicki O, Lucchi G, et al. Inter-individual variability of protein patterns in saliva of healthy adults. J Proteomics. 2009;72:822–830. 19470415 10.1016/j.jprot.2009.05.004

[39] Gonçalves LDR, Soares MR, Nogueira FCS, et al. Comparative proteomic analysis of whole saliva from chronic periodontitis patients. J Proteomics. 2010;73:1334–1341. 20215060 10.1016/j.jprot.2010.02.018

[40] Gutiérrez A, Cerón JJ, Razzazi-Fazeli E, et al. Influence of different sample preparation strategies on the proteomic identification of stress biomarkers in porcine saliva. BMC Vet Res. 2017;13:1–11. 29202764 10.1186/s12917-017-1296-9 PMC5716369

[41] Ploypetch S, Roytrakul S, Jaresitthikunchai J, et al. Salivary proteomics of canine oral tumors using MALDI-TOF mass spectrometry and LC-tandem mass spectrometry. PLoS One. 2019;14:1–21. 10.1371/journal.pone.0219390 PMC6638856 31318878

[42] Costa MM, Martin H, and Estellon B, et al. Exploratory study on application of MALDI-TOF-MS to detect SARS-CoV-2 infection in human saliva. J Clin Med. 2021. 10.3390/jcm11020295 PMC8781148 35053990

